# Tumor perfusion evaluation using dynamic contrast-enhanced ultrasound after electrochemotherapy and IL-12 plasmid electrotransfer in murine melanoma

**DOI:** 10.1038/s41598-021-92820-w

**Published:** 2021-06-29

**Authors:** Maja Brloznik, Nina Boc, Maja Cemazar, Gregor Sersa, Masa Bosnjak, Simona Kranjc Brezar, Darja Pavlin

**Affiliations:** 1 Clinic for Small Animals, Veterinary Faculty, University of Ljubljana, Gerbičeva 60, Ljubljana, Slovenia; 2 Institute of Oncology Ljubljana, Zaloška 2, Ljubljana, Slovenia; 3 Faculty of Health Sciences, University of Primorska, Polje 42, Izola, Slovenia; 4 Faculty of Health Sciences, University of Ljubljana, Zdravstvena 5, Ljubljana, Slovenia; 5 Faculty of Medicine, University of Ljubljana, Vrazov trg 2, Ljubljana, Slovenia

**Keywords:** Biophysics, Cancer, Medical research

## Abstract

Electrochemotherapy with bleomycin (ECT BLM) is an effective antitumor treatment already used in clinical oncology. However, ECT alone is still considered a local antitumor therapy because it cannot induce systemic immunity. When combined with adjuvant gene electrotransfer of plasmid DNA encoding IL-12 (GET pIL-12), the combined therapy leads to a systemic effect on untreated tumors and distant metastases. Although the antitumor efficacy of both therapies alone or in combination has been demonstrated at both preclinical and clinical levels, data on the predictors of efficacy of the treatments are still lacking. Herein, we evaluated the results of dynamic contrast-enhanced ultrasound (DCE-US) as a predictive factor for ECT BLM and GET pIL-12 in murine melanoma. Melanoma B16F10 tumors grown in female C57Bl/6NCrl mice were treated with GET pIL-12 and ECT BLM. Immediately after therapy, 6 h and 1, 3, 7 and 10 days later, tumors were examined by DCE-US. Statistical analysis was performed to inspect the correlation between tumor doubling time (DT) and DCE-US measurements using semilinear regression models and Bland–Altman plots. Therapeutic groups in which DCE-US showed reduced tumor perfusion had longer tumor DTs. It was confirmed that the DCE-US parameter peak enhancement (PE), reflecting relative blood volume, had predictive value for the outcome of therapy: larger PE correlated with shorter DT. In addition, perfusion heterogeneity was also associated with outcome: tumors that had more heterogeneous perfusion had faster growth, i.e., shorter DTs. This study demonstrates that DCE-US can be used as a method to predict the efficacy of electroporation-based treatment.

## Introduction

Electrochemotherapy with bleomycin (ECT BLM) has been shown to be an effective antitumor treatment in numerous clinical trials^1–6^. It is a local treatment that combines the use of electroporation (EP) and chemotherapeutic agents and has a dual effect on tumor cells and the vasculature, resulting in an antitumor effect in various solid tumors^5–7^. Many studies have indicated the contribution of the immune system to the efficiency of ECT, which induces immunogenic cell death by activating molecular signals called danger-associated molecular patterns (DAMPs) that strengthen the innate immune cells that drive the production of specific antitumor immunity^7–12^. However, ECT alone is unable to induce systemic immunity, whereas ECT in combination with adjuvant gene electrotransfer (GET) results in an 'abscopal effect' on untreated distant metastases^7,8^. Recent preclinical and clinical studies have shown that the effect of ECT can be potentiated by GET using plasmid DNA encoding IL-12 (GET pIL-12)^7,8,10,13–17^.

Based on the results of clinical trials^1–4^, ECT BLM has been listed in several national and international guidelines as an effective treatment option for various tumor types in human^5,6,18^ and veterinary oncology^19^. Clinical trials using GET pIL-12 have been performed in human^20–22^ and veterinary patients^7,13,14,23–25^, but standard operating procedures for GET pIL-12 are still pending.

High-amplitude electrical pulses induce a local reduction in blood flow or 'vascular lock' characterized by vasoconstriction and increased wall permeability of small blood vessels^10,26–29^. Furthermore, the vascular effects of EP-based therapies are not only caused by the direct effect of electrical pulses on cells but are further enhanced by the use of chemotherapeutic agents in ECT treatment. The effect on perfusion lasts longer in ECT than in EP alone and longer in tumors than in healthy tissues, which is referred to as the 'vascular disrupting effect'^10,26,29^.

Despite the known clinical efficacy of combined treatments of ECT and GET, there is still a lack of data on parameters that could predict outcome of these treatments. One of the possible methods to determine the predictive factors of therapy is dynamic contrast-enhanced ultrasound (DCE-US), a noninvasive method used to assess tissue perfusion at the capillary level, which correlates with histological results of vascular density in preclinical^30–32^ and clinical studies^33–36^. DCE-US has been used to predict the efficacy of various antiangiogenic treatments in both human^37–42^ and preclinical studies^31^.

Current ultrasound contrast agents are gas-filled microbubbles: a relatively insoluble gas core is stabilized within a phospholipid shell that provides relative stability in plasma for several minutes^43^. Microbubbles are 1–3 μm in diameter, smaller than mouse red blood cells, which are approximately 6 μm in diameter. Microbubbles pass freely throughout the pulmonary and systemic circulation, and capillary filling leads to diffuse enhancement of the perfused tissue. To perform DCE-US, contrast-specific software with a low mechanical index (MI) is required to visualize microbubbles without destroying them. Unlike tissue, microbubbles have a nonlinear response when used with a low MI^43,44^.

Investigating perfusion is an attractive method for predicting antitumor effects, especially in antiangiogenic treatments, e.g., ECT combined with GET. In combined ECT and GET treatments, it is reasonable to assume that repetition of the therapy is appropriate if the therapy does not result in the expected 'vascular lock' and/or the antiangiogenic effects are not evident in the days after treatment.

This study aimed to evaluate the results of DCE-US, including tumor perfusion heterogeneity, as predictive factors of ECT BLM and GET pIL-12 on murine B16F10 melanoma growth.

## Results

### Tumor growth delay (GD) after ECT BLM and GET pIL-12

For the DCE-US studies, murine B16F10 melanoma tumors treated with ECT BLM and GET pIL-12 were used as a model, which has previously been shown to be feasible for studying the antitumor effect of EP-based treatments.

**Figure 1:**
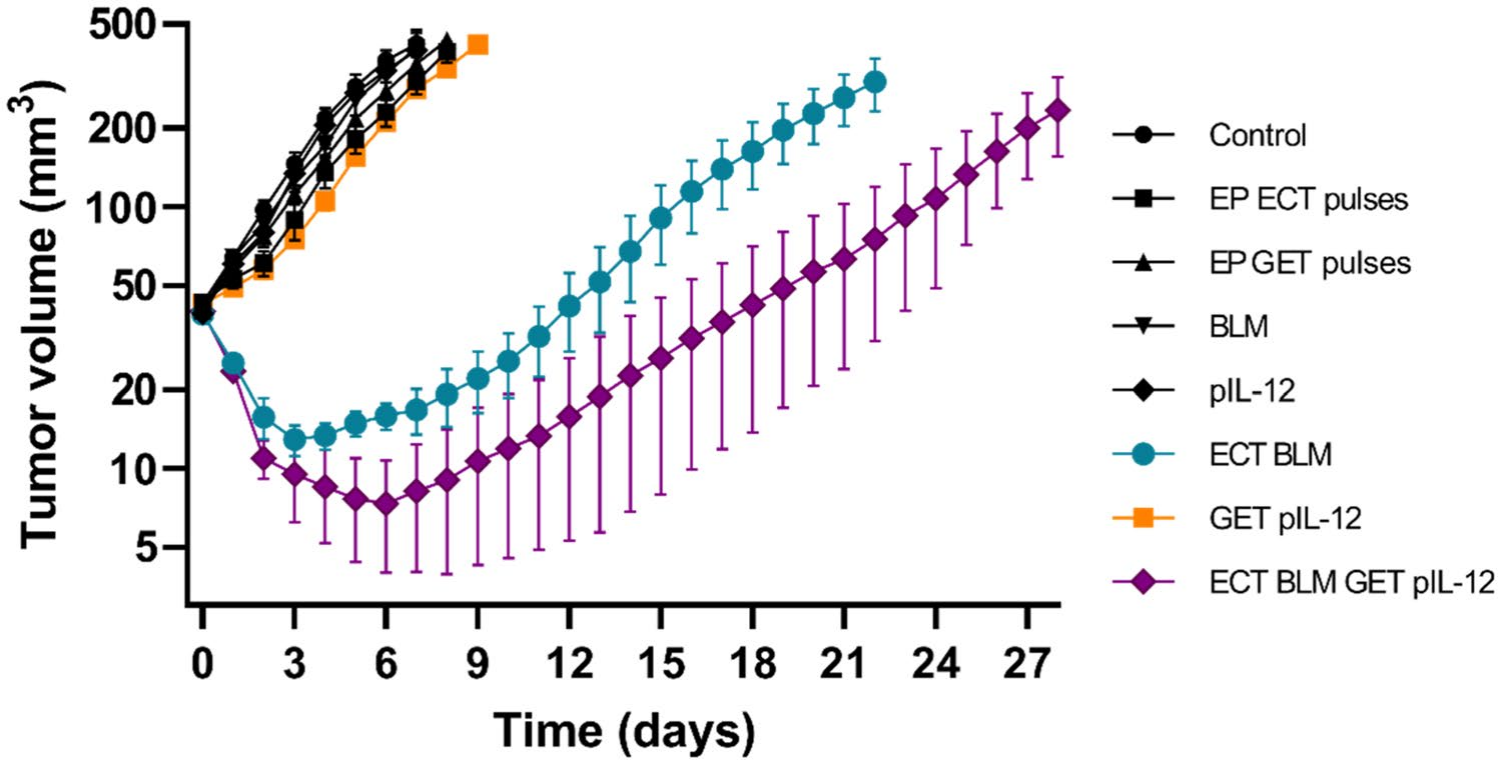
Mean tumor volume in mouse melanoma B16F10 cells treated with electrochemotherapy with bleomycin and gene electrotransfer of plasmid DNA encoding IL-12. Data are presented as the mean and standard error of the mean (SE) (n = 66). BLM = bleomycin, 7.5 µg/mouse; ECT = electrochemotherapy; EP = electric pulses; GET = gene electrotransfer; pIL-12 = plasmid DNA encoding mouse interleukin-12. Note that significant tumor growth delay was observed in the mice treated with ECT BLM and ECT BLM combined with GET pIL-12. In GET pIL-12 and other groups (untreated control and pertinent controls) no antitumor efficiacy was observed.

Monotherapies, i.e., pIL-12, BLM and application of electrical pulses (EP ECT; EP GET), and combined treatment with GET pIL-12 had no significant antitumor effect and resulted in tumor GD of up to only 1.9 days compared to untreated tumors (Fig. 1, Supplementary Table S1). However, the growth of B16F10 melanoma tumors was significantly delayed when ECT BLM was performed either alone or in combination with GET pIL-12. Tumor GD after ECT BLM treatment was 14 days, and after ECT BLM GET, pIL-12 was 26.7 days.

### DCE-US results

To monitor the effect of treatment on tumor perfusion, DCE-US measurements were performed at different time points after therapy.

There was no correlation between tumor growth and blood flow parameters (AT, AS, TTP, DT/2, DS) or AUC blood volume parameters. Only a correlation between tumor growth and PE was confirmed; therefore, only PE was further analyzed and is reported below.

Of note, mice in the untreated CTRL and pertinent CTRL groups were not measured on days seven and ten because they were humanely sacrificed on day six due to disease burden.

#### DCE-US results in the treatment groups

**Figure 2:**
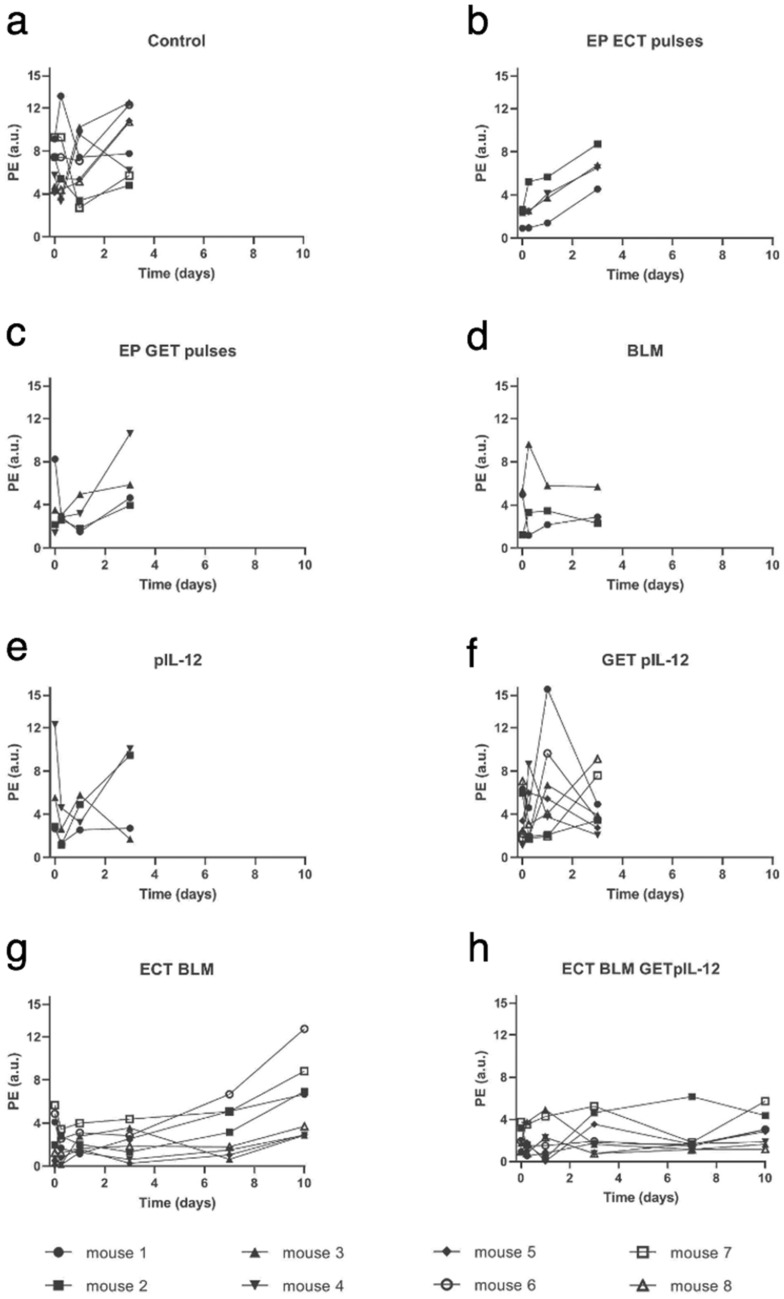
Peak enhancement on different days in mouse melanoma B16F10 cells treated with electrochemotherapy with bleomycin and gene electrotransfer of plasmid DNA encoding plasmid IL-12. Based on dynamic contrast-enhanced ultrasound (DCE-US) measurements, peak enhancement was calculated as the difference between peak intensity and base intensity. Note that mice in the control and pertinent control groups were not measured on days 7 and 10 because they were humanely sacrificed on day 6 due to disease burden. BLM = bleomycin, 7.5 µg/mouse; ECT = electrochemotherapy; EP = electric pulses; GET = gene electrotransfer; pIL-12 = plasmid DNA encoding mouse interleukin-12; PE = peak enhancement. Note that PE values are lower for ECT BLM group (G) and ECT BLM GET pIL-12 group (H) than for other groups.

Immediately after therapy, the PE values in the groups in which electrical pulses for ECT were applied alone (EP ECT pulses; 2.0 ± 0.8) combined with an intratumoral injection of BLM (ECT BLM, 2.4 ± 0.8) or combined with an intratumoral injection of BLM and GET pIL-12 (ECT BLM GET pIL-12;1.7 ± 0.5) were lower than those in the untreated CTRL group (CTRL; 6.5 ± 0.7) (Fig. 2, Supplementary Table S2). This result was confirmed by DCE-MRI imaging of the tumors, which demonstrated decreased perfusion in the tumors immediately and 6 h after ECT compared to untreated CTRL tumors (Supplementary Fig. S1).

Furthermore, six hours, one day and three days after therapy, the PE values were significantly lower in the ECT BLM (1.6 ± 0.4, 2.0 ± 0.3, 2.2 ± 0.5) and ECT BLM GET pIL-12 groups (2.2 ± 0.7, 2.0 ± 0.6, 2.7 ± 0.7) than in the untreated CTRL group (6.5 ± 1.2, 6.2 ± 1.0, 8.4 ± 1.3).

**Figure 3:**
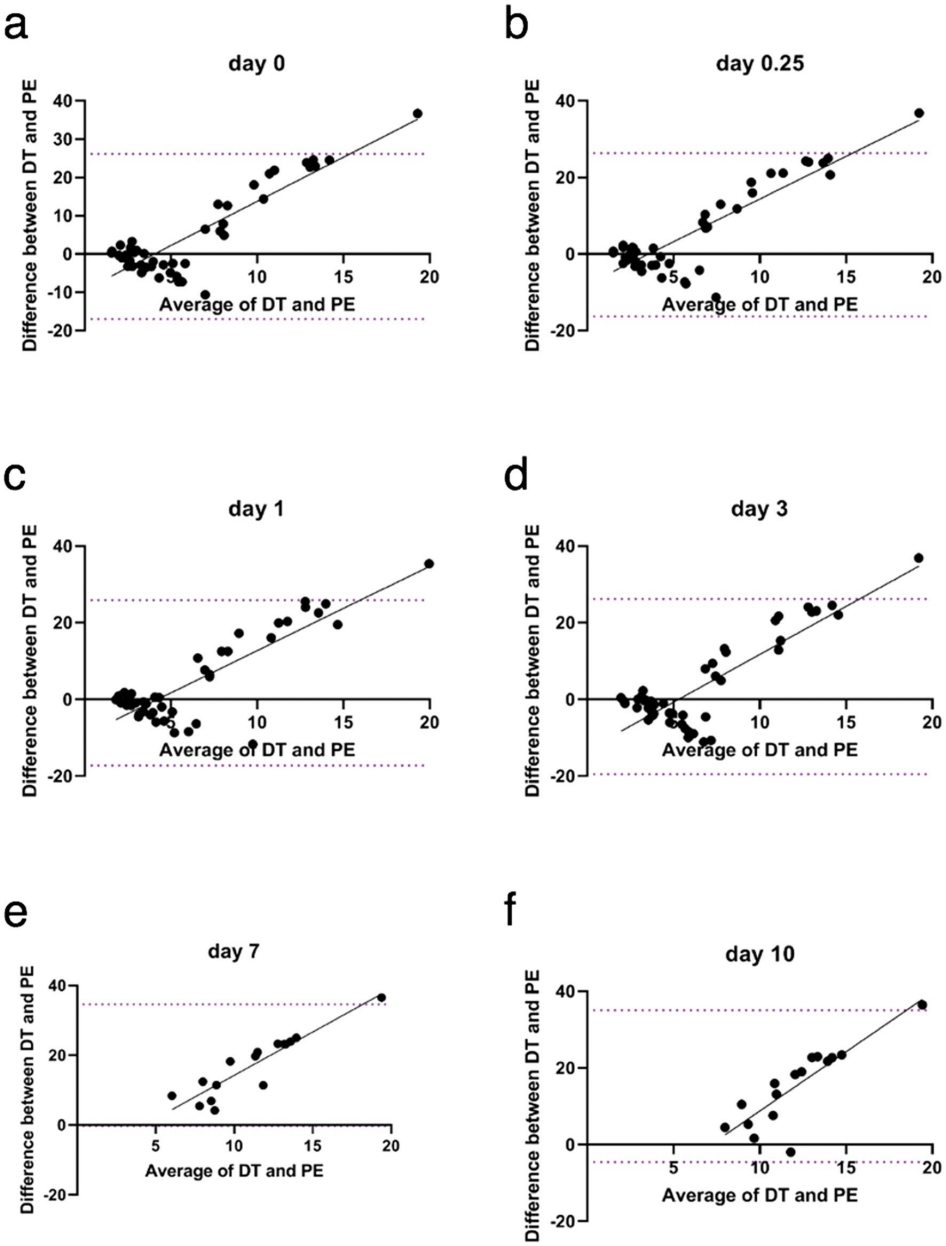
Bland–Altman plot of differences between tumor doubling time and peak enhancement vs. the average of the two measurements at different time points (a – f). Dotted lines represent the 95% confidence interval. Gray lines are for visual inspection only. BLM = bleomycin, 7.5 µg/mouse; DT = doubling time; ECT = electrochemotherapy; EP = electric pulses; GET = gene electrotransfer; pIL-12 = plasmid DNA, encoding mouse interleukin-12; PE = peak enhancement. Note that on days 7 and 10, only mice in the therapeutic groups (ECT BLM and ECT BLM combined with GET pIL-12) were measured because mice in the control groups were humanely sacrificed on day 6 due to the disease burden. Each group consisted of 3–8 animals.

The Bland–Altman plots presented in Fig. 3 show good agreement between PE and DT. The confidence intervals were narrower on days 1, 7 and 10.

#### DCE-US results as predictive factors for response to treatment

**Figure 4:**
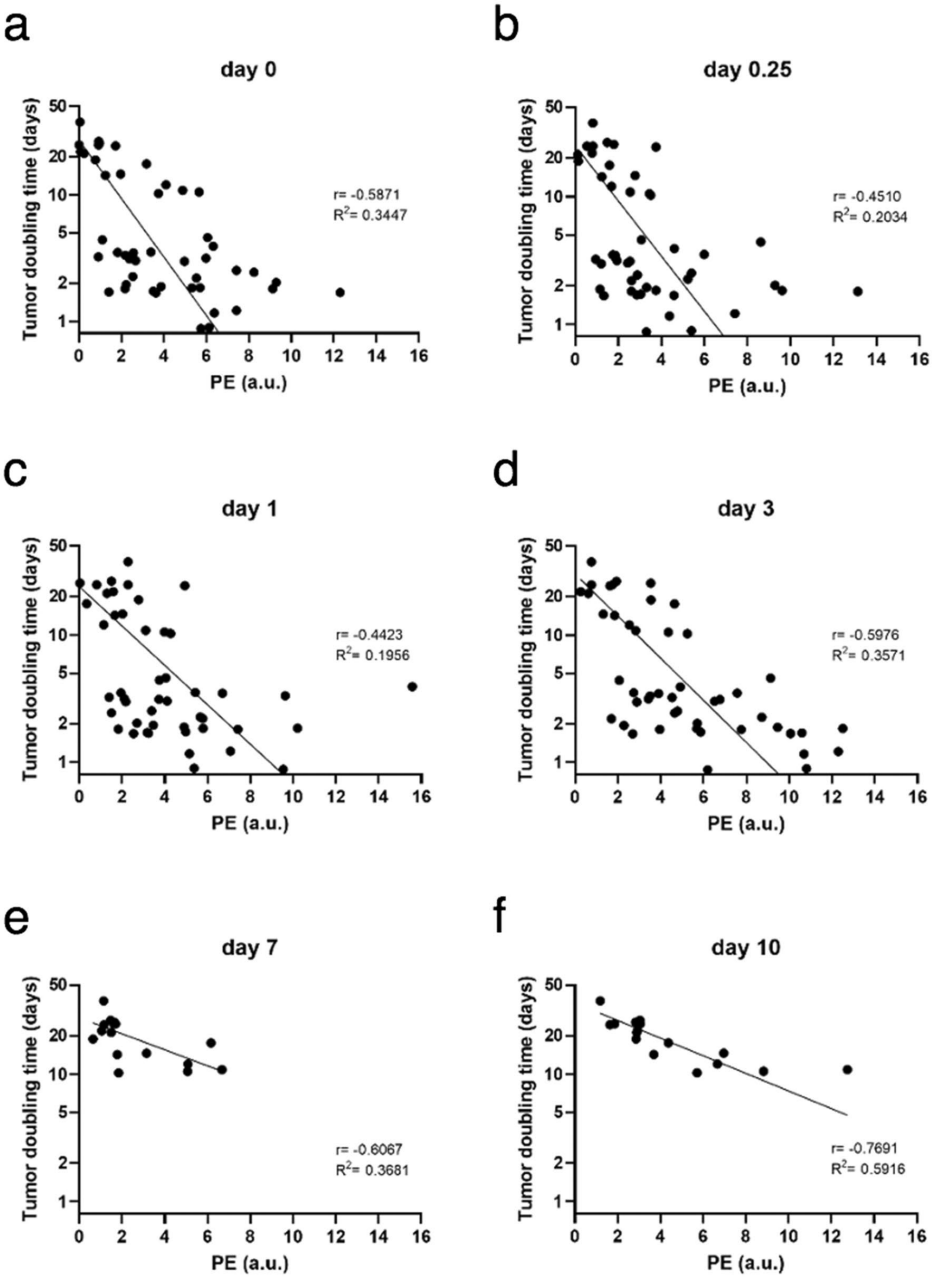
Correlation between peak enhancement and logarithmically transformed doubling time in B16F10 melanoma at different time points (a – f) after treatment with bleomycin electrochemotherapy and gene electrotransfer of a plasmid encoding mouse interleukin-12. BLM = bleomycin, 7.5 µg/mouse; DT = doubling time; ECT = electrochemotherapy; EP = electric pulses; GET = gene electrotransfer; pIL-12 = plasmid DNA encoding mouse interleukin-12; PE = peak enhancement. On days 0, 0.25, 1 and 3, DCE-US was performed in all the treatment groups: control, electroporation (EP) of tumors or the skin using a different set of pulses, intratumoral injection of BLM, intradermal injection of pIl-12, ECT BLM, GET pIl-12, and ECT BLM combined with GET pIL-12. Note that on days 7 and 10, only mice in the therapeutic groups (ECT BLM and ECT BLM combined with GET pIL-12) were measured because mice in the control groups were humanely sacrificed on day 6 due to the disease burden.

**Figure 5:**
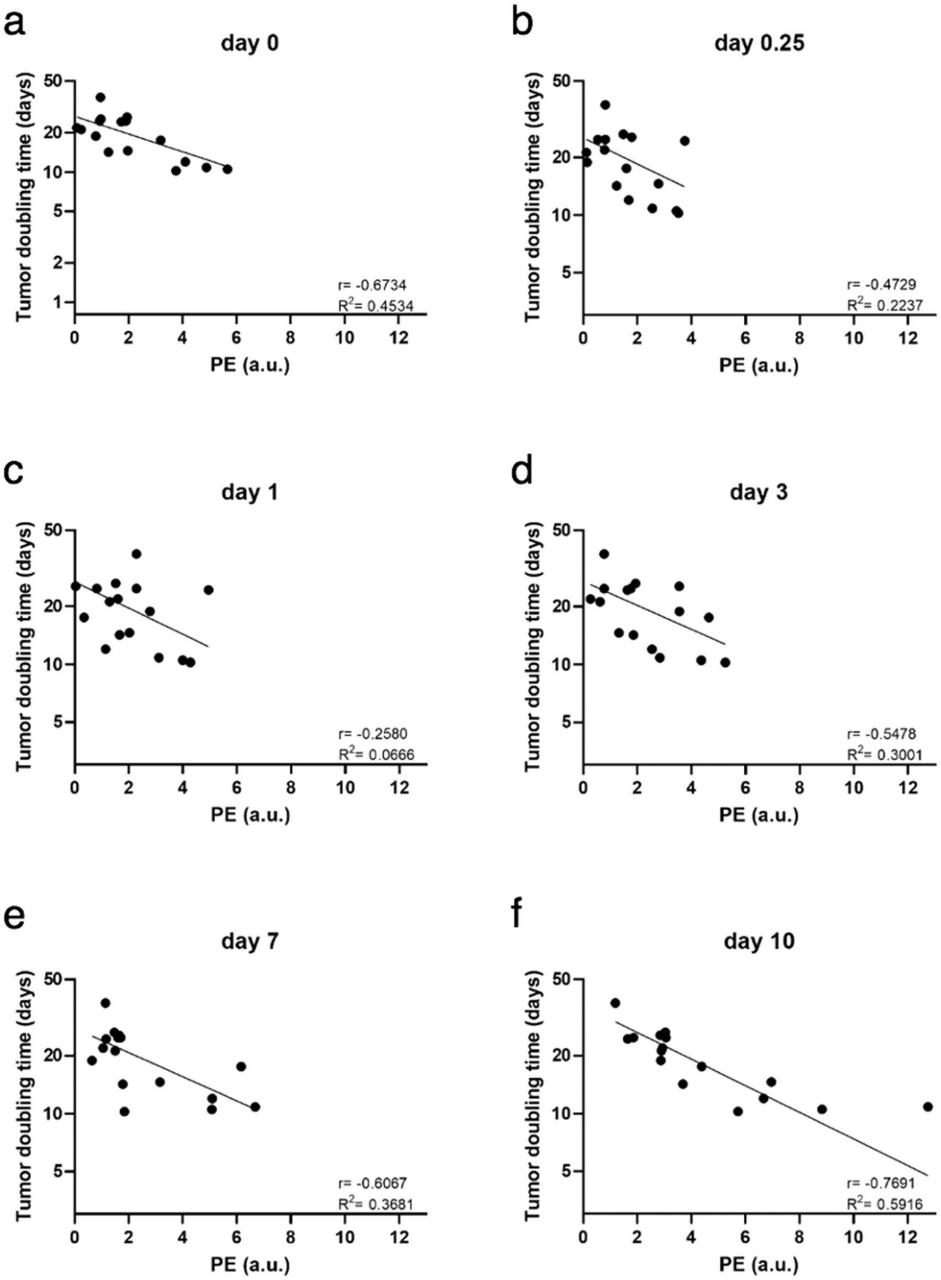
Correlation between peak enhancement and logarithmically transformed doubling time in B16F10 melanoma at different time points (a–f) after treatment with bleomycin electrochemotherapy and gene electrotransfer of plasmid encoding mouse interleukin-12. BLM = bleomycin, 7.5 µg/mouse; DT = doubling time; ECT = electrochemotherapy; EP = electric pulses; GET = gene electrotransfer; pIL-12 = plasmid DNA encoding mouse interleukin-12; PE = peak enhancement. Note that only data for mice in the therapeutic groups (ECT BLM and ECT BLM combined with GET pIL-12) are presented.

The association between PE and logarithmically transformed DT for all groups is shown in Fig. 4. Note that mice with the most advanced tumors (the shortest DT values) were excluded from the analyses early (no dots for small DT values at days 7 and 10). Negative regression coefficients (r) indicated that the larger the PE of a mouse was, the lower the expected DT. Statistically significant Pearson coefficients showed a weaker correlation at the second and third time points of the DCE-US measurements (6 h and 1 day after therapy) (− 0.5 and − 0.4, respectively) and a stronger correlation immediately after treatment and on days 3 and 7 (− 0.6) and the strongest correlation on day 10 (− 0.8) (Supplementary Table S3). The association between DCE-US PE and logarithmically transformed DT of mice in the two therapeutic groups showed the weakest correlation on day 1 (r = − 0.3) and the strongest correlation on days 0 and 10 (r = − 0.7 and r = − 0.8, respectively) (Fig. 5, Supplementary Table S4).

#### DCE-US results for heterogeneity of tumor perfusion

**Figure 6:**
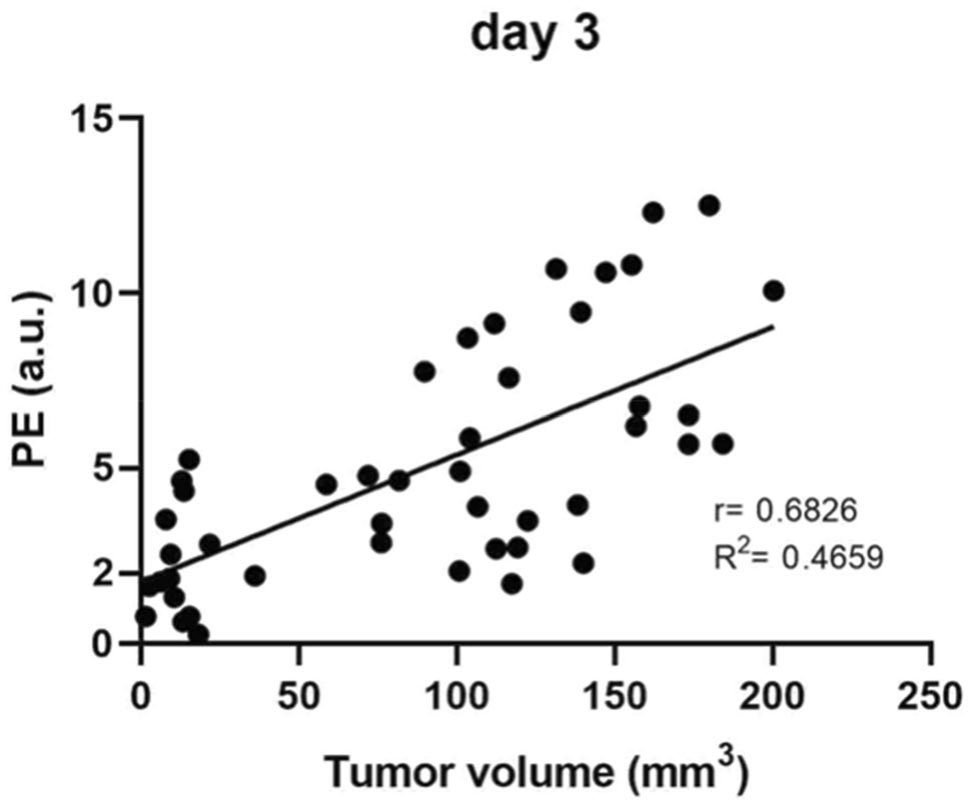
Correlation between peak enhancement and tumor volume at day 3 in melanoma B16F10 cells after treatment with bleomycin electrochemotherapy and gene electrotransfer of a plasmid DNA encoding mouse interleukin-12. Dots represent data obtained from all 47 animals in the experiment. PE = peak enhancement.

**Figure 7:**
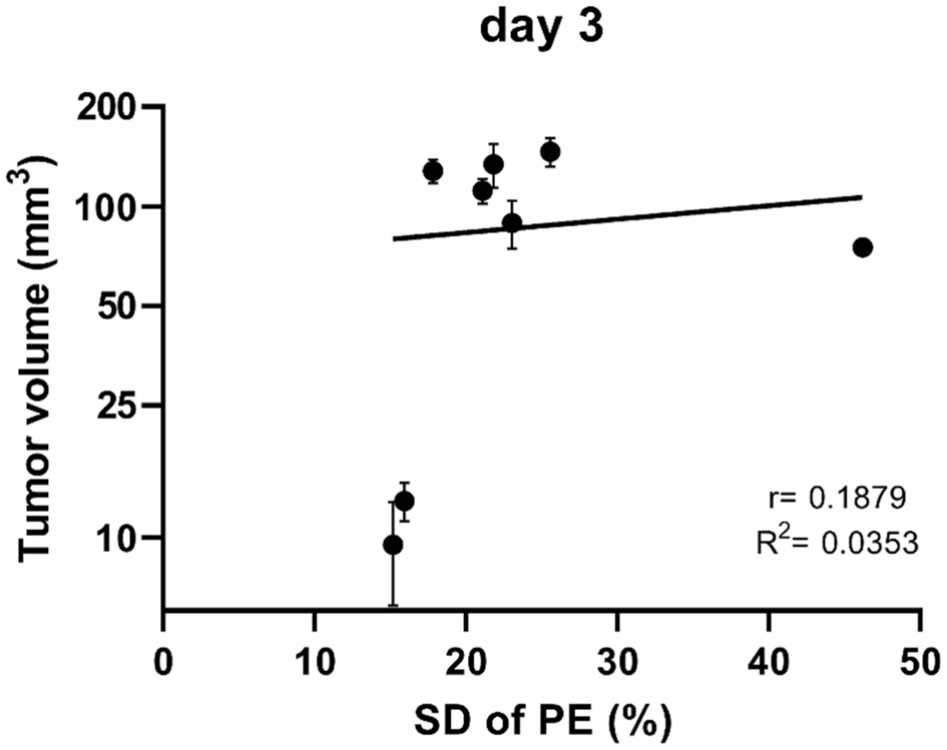
Correlation between tumor volume and the standard deviation of peak enhancement on day 3 after treatment with bleomycin electrochemotherapy and gene electrotransfer of plasmid DNA encoding mouse interleukin-12. PE = peak enhancement; SD = standard deviation. Data were obtained from 66 animals included in the experiment. Note that the larger the tumor volume is, the larger the SD of PE.

Perfusion curves for different ROIs of the same tumor showed that tumors larger than 40 mm^3^ were often heterogeneously perfused; note the different perfusion curves for different ROIs in Supplementary Fig. S2. In larger tumors, the PE values were higher (Fig. 6), and heterogeneous perfusion was more pronounced with increasing tumor volume in all the treatment groups; the SD of PE versus tumor volume showed that the larger the tumor volume, the greater the SD of PE (Fig. 7). In addition, mice in the same treatment group exhibited heterogeneous PE values; the SD of PE was large (± 25.9% on average and up to ± 52.6%). It should also be noted that the variability of perfusion for the same tumor on different days was high (Fig. 2).

**Figure 8:**
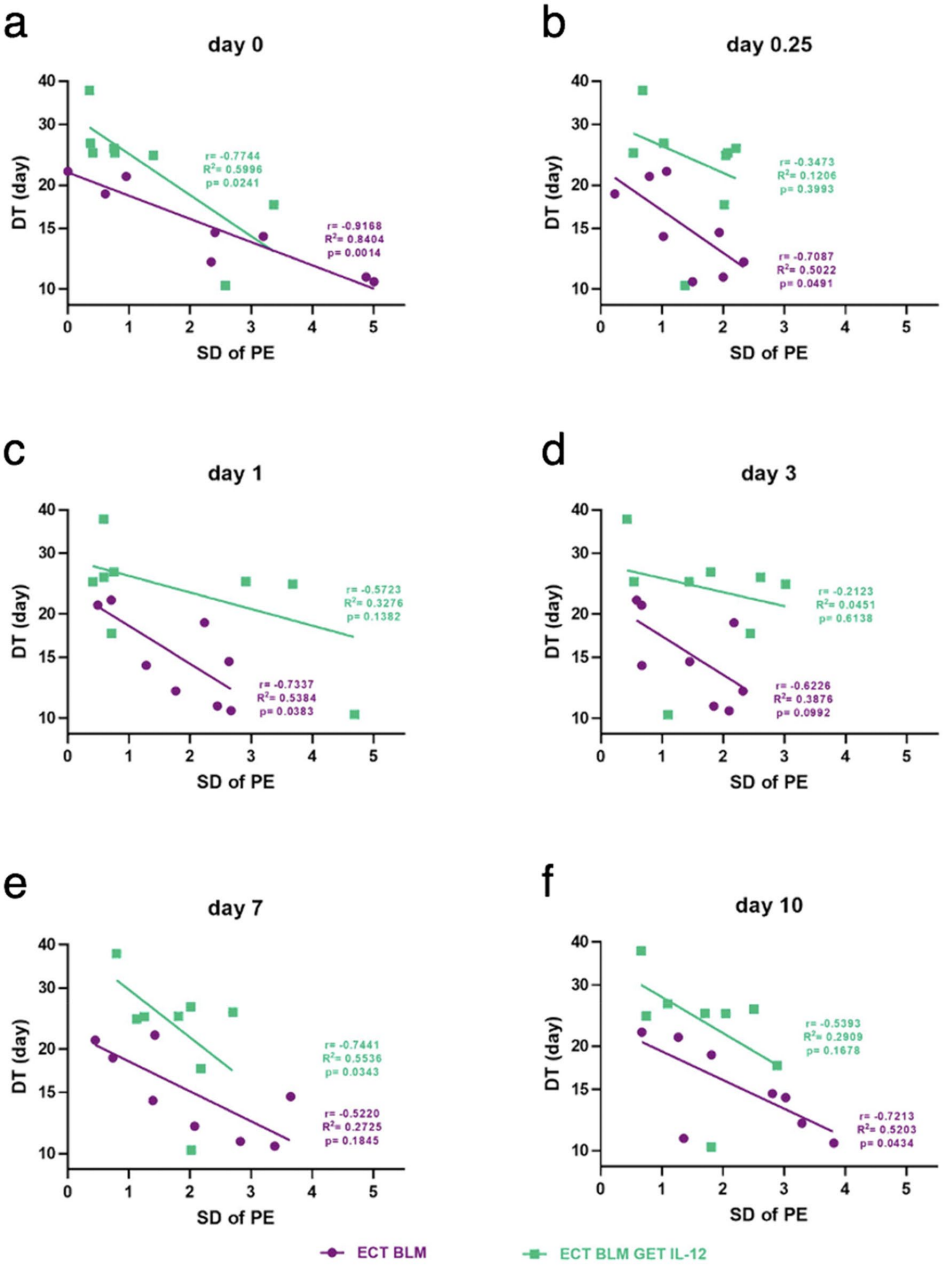
Correlation between the standard deviation of peak enhancement for different regions of interest of a tumor and DT in B16F10 melanoma at different time points (a – f) after treatment with bleomycin electrochemotherapy and gene electrotransfer of a plasmid encoding mouse interleukin 12. BLM = bleomycin, 7.5 µg/mouse; DT = doubling time; ECT = electrochemotherapy; EP = electric pulses; GET = gene electrotransfer; pIL-12 = plasmid DNA encoding mouse interleukin-12; PE = peak enhancement; ROI = region of interest; SD = standard deviation. The presented data were obtained from tumors treated with ECT BLM and ECT BLM combined with GET pIL-12 (n = 16).

The SD of PE for ROIs in the same tumor was compared with DT (Fig. 8); the greater the perfusion heterogeneity (i.e., SD of PE), the shorter the DT. In the ECT BLM group, there was a significant correlation for measurements immediately after therapy (p = 0.0014), 6 h after therapy (p = 0.049), and on day 1 (p = 0.038) and day 10 (p = 0.043). Negative regression coefficients (r) indicated that the larger the SD of PE of a tumor the shorter the expected DT. For ECT BLM GET pIL-12, the correlation was significant only on days 0 and 7 (p = 0.024 and p = 0.034, respectively), with Pearson coefficients of − 0.8 and − 0.7, respectively.

## Discussion

Our study confirmed that DCE-US can be used to predict the outcome of EP-based treatments in a mouse melanoma model. Our study also shows that tumor perfusion heterogeneity correlates with treatment outcome. The ultrasonographically determined mean PE values were significantly lower in the groups with shorter tumor DT, and the larger the PE of a mouse, the slower the tumor growth. In our study, the correlation between PE and DT increased with time, with the highest Pearson coefficient at day 10, when only mice of both therapeutic groups were still alive. Immediately after therapy, decreased mean PE values were obtained for the three groups treated with EP for ECT. This local blood flow altering effect or ‘vascular lock’ characterized by vasoconstriction and increased wall permeability of small blood vessels is induced by high-voltage electrical pulses^10,26–29,45,46^, and they were used only in three groups and not in the other groups. Blood flow is slowly restored after EP and approaches the previous flow within 24 h^10,27,29^. The vascular effects of EP are exacerbated by the use of chemotherapeutic agents that are cytotoxic to neoplastic endothelial cells, and this effect prolongs reduced blood flow^26,27^. The results of DCE-US as predictors of EP-based treatments and tumor heterogeneity correlated with tumor growth have not been previously reported. However, for the clinical use of DCE-US for tumor perfusion and antitumor effect prediction, further studies are needed to determine appropriate cutoff values for DCE-US parameters and tumor perfusion conditions that take into account tumor size, perfusion heterogeneity and others to effectively decide on prognosis and the need for repeated therapy. The results of our study are consistent with preclinical studies in which DCE-US results were predictive of treatment success with cisplatin and thalidomide^30,31^ and with human clinical studies in which DCE-US results were predictive of antiangiogenic chemotherapeutic treatment^37–42^, whereas DCE-US had no predictive value for the radiation outcome in canine tumors^47,48^. Our results are encouraging; previous methods for assessing vascularity in tumors after EP-based treatment are mostly invasive, e.g., immunohistochemistry, and have not been shown to be predictive of EP-based treatment success. In contrast, DCE-US is noninvasive and repeatable and therefore could be used for longitudinal tumor monitoring and decision making when using EP-based therapy in a patient. If therapy does not result in the expected ‘vascular lock’ or decreased perfusion in the following days, as shown in our study, then therapy should be repeated if needed.

The concept of tumor heterogeneity has not yet been incorporated into current clinical oncology practice or the evidence-based cancer treatment paradigm^48^. Nevertheless, there is evidence that heterogeneous functional/biological characteristics of tumors significantly influence treatment outcomes^49^. Perfusion heterogeneity is common in solid aggressive tumors^50,51^, and this was also observed for melanoma tumors in this study, with perfusion heterogeneity becoming more pronounced with increasing tumor volume across all treatment groups. The heterogeneity of tumors could be explained by impaired mechanisms regulating vascular growth. Tumor vessels are abnormal—immature—with chaotic tumor vascular networks and only intermittent flow through many vascular branches^52,53^. Such an aberrant vasculature was observed by DCE-US in our study; in general, the periphery of the tumors was more perfused, and rapid wash-in and wash-out were observed. Moreover, mice of the same group exhibited heterogeneous PE values with high standard deviations (up to ± 41.5%). In addition, we demonstrated a correlation between perfusion heterogeneity and tumor response; the SD of PE for different ROIs of the tumor was correlated with DT; the more heterogeneously perfused the tumors were, the shorter the DT. This was particularly true for the ECT BLM group, in which we observed a statistically significant correlation between the two parameters throughout the observation period. In contrast, in the group that received GET in addition to ECT, there were only two statistically significant time points (immediately after therapy and on day 7). This could mean that we affected the tumor vasculature and perfusion heterogeneity when GET p-IL12 was added to ECT BLM. Similar to our results, perfusion heterogeneity evaluated by DCE-MRI in cervical cancer treated with radio- and chemotherapy^49^ and by DCE-CT in hepatic neoplasia treated with antiangiogenic therapy has been shown to correlate with local tumor control and survival^54^. The first mentioned study showed an opposite result to ours, reporting that decreased perfusion correlated with a worse treatment outcome for radiotherapy in cervical cancer because the response to radiation therapy is lower in parts of the tumor that are hypoxic due to decreased perfusion^49^. The results of the second study were similar to ours, in which a negative percentage change in the heterogeneity parameter correlated with better treatment outcomes of hepatic neoplasia treated with antiangiogenic therapy^54^. Perfusion heterogeneity may lead to unfavorable drug delivery and an increasing hypoxic environment that accelerates cancer progression^54^. Although a heterogeneous response is typical of many cancer treatments, the treatment regimen remains largely uniform, which may be why treatment failure is so common in different cancers where recommended guidelines for the most appropriate treatment have been followed^49^. Predicting treatment outcome is challenging when performed as early as possible during treatment, when targeted adjustments to therapy are generally more effective^49^. Our tumor heterogeneity results show that not only tumor perfusion intensity but also perfusion heterogeneity can be used to assess treatment outcome in ECT BLM treatment; if tumors are still heterogeneous after treatment, then repeated therapy should be considered. In the combined ECT BLM GET pIL-12 treatment, perfusion heterogeneity correlated with tumor growth at two time points only; therefore, further studies are needed to evaluate whether GET p-IL12 affects tumor vasculature in such a way that tumor perfusion heterogeneity is less predictive of tumor growth.

A major limitation of our study was that there were no complete responders. This could be due to the low BLM dose; we used 7.5 μg of BLM per mouse, whereas in previous studies, higher dosages were used^26^. This lower BLM dose was chosen to avoid too many CRs due to the high antitumor effect of ECT BLM. Our goal was to achieve a broad spectrum of different responders: CR, partial response, response with stable disease, and response with varying progress. The pharmacokinetics of BLM depend on tumor vascularity, which affects the accumulation and distribution of the drug in the tumor: the more vascularized the tumors are with small vessels, the better the effect of ECT, and conversely, when the vessels are larger, the washout of the chemotherapeutic drug is greater and the effect of ECT is lower^55^. In addition, no significant antitumor effect was observed when treated with peritumoral GET p-IL12. This result is in contrast to studies reporting the antitumor efficacy of intratumoral GET p-IL12^56^ and GET with control plasmid DNA^9,11^. The most likely reasons for the lower antitumor efficacy of GET p-IL12 in our study are the peritumoral application of pIL-12, a different dose of pIL-12 and/or a different protocol of application of electrical pulses (56). In our study, the GD of the two therapeutic groups was 15 days and 28 days, which is consistent with preclinical and clinical studies reporting the efficacy of ECT with BLM^1–5^ and potentiation of ECT with GET^6–8,13,14^.

This study demonstrates that DCE-US, a simple, noninvasive, safe, and inexpensive method of tissue perfusion, can be used to predict tumor growth after EP-based therapy. The information obtained with DCE-US is readily available and DCE-US can be easily repeated during therapy. Due to its potential value as a prognostic and predictive factor of disease, further preclinical and clinical studies are needed to confirm these preclinical study results in mice. If confirmed, the results of DCE-US can be used to predict the course of the disease and thus can even be used as a decision-making tool for planning repeated therapy or different treatment combinations in individual patients.

## Methods

The study was carried out in compliance with the ARRIVE guidelines.

### Tumor cells

Melanoma B16F10 cells were cultured in Advanced Minimal Essential Medium (AMEM) containing 5% fetal bovine serum (FBS) (Gibco, Fisher Scientific, Waltham, MA, USA), 10 mM/L L-glutamine GlutaMAX (Gibco, Fisher Scientific, Waltham, MA, USA), 100 U/mL penicillin (Gruenenthal, Aachen, Germany) and 50 mg/mL gentamicin (Krka, Novo Mesto, Slovenia) in an incubator humidified with 5% CO_2_ at 37 °C. At 80% confluence, trypsinization was performed with 0.25% trypsin/EDTA in Hank’s buffer. Cells were then washed with AMEM containing 5% FBS and collected by centrifugation. Tumors were induced on the backs of mice by a subcutaneous injection of 100 µl of a B16F10 cell suspension containing one million cells prepared in a 0.9% NaCl solution (B Braun, Melsungen AG, Melsungen, Germany).

### Animals

Female C57Bl/6NCrl mice (Envigo RMS SrL, San Pietro al Natisone, Italy) that were 7 weeks old and weighed 18–20 g were housed under specific pathogen-free conditions at a temperature of 20–24 °C, a relative humidity of 55 ± 10%, a 12-h light–dark cycle and ad libitum food and water. All procedures were performed according to the guidelines for animal experiments of the EU Directive (2010/63/EU). Official approval was granted by the Veterinary Administration of the Ministry of Agriculture, Forestry and Food of the Republic of Slovenia (No. U34401-1/2015/43).

### BLM

BLM (Bleomycin medac, Medac, Wedel, Germany, BLM) was dissolved to 3 mg/mL in sterile water (B Braun, Melsungen AG, Melsungen, Germany), aliquoted and frozen at -20 °C until use. A fresh BLM solution of 0.375 mg/mL in 0.9% NaCl was prepared (B Braun, Melsungen AG, Melsungen, Germany) before intratumoral injection (7.5 μg of BLM in 20 μl).

### IL-12 plasmid

The pORF-mIL-12-ORT (pIL-12) plasmid, which encodes the mouse gene IL-12 and lacks an antibiotic resistance gene, was used. Construction of the plasmid has been previously described^57^. The plasmid was isolated from a bacterial culture using an EndoFree Plasmid Mega kit (Qiagen, Hilden, Germany) and diluted to a concentration of 0.625 mg/mL in endotoxin-free water (Qiagen, Hilden, Germany). Purity and yield were determined spectrophotometrically (Epoch Microplate Spectrophotometer, Take3™ microvolume plate, BioTek, Bad Friedrichshall, Germany). Before experiments, the concentration and identity of the plasmid were confirmed by restriction analysis on an electrophoresis gel.

### Experimental design and DCE-US examinations

The experiment began when tumors reached approximately 40 mm^3^ (6 × 6 × 2 mm in orthogonal diameters) (considered day 0). Mice were randomly divided into eight groups with 8–12 mice each. There was no tumor manipulation in the control group (CTRL). There were two groups in which only different electrical pulses were applied: in the EP ECT group, electrical pulses were applied for ECT (8 square electrical pulses with a voltage-distance ratio of 1300 V/cm, a pulse duration of 100 μs and a frequency of 1 Hz), and in the EP GET group, electrical pulses were applied in GET to the skin (12 electrical pulses, 150 ms long with a voltage-distance ratio of 170 V/cm at 2.82 kHz). The selection of the voltage, duration and frequency of the electrical pulses was based on ECT^2,3,5–7,17,18,58^ and GET guidelines^15,19–24,59,60^. The electrical pulses for EP ECT were generated using the electrical pulse generator ELECTRO CELL B10 HVLV (Betatech, L’Union, France) and delivered through two parallel stainless-steel electrodes 6 mm apart. After delivery of 4 pulses, the electrodes were rotated 90°, and four more electrical pulses were applied to expose the entire tumor to the electrical pulses. The electrical pulses for EP GET were generated using the Cliniporator electrical pulse generator (IGEA S.p.A., Carpi, Italy); 12 electrical pulses were delivered to the skin through a noninvasive multielectrode array (MEA) consisting of six spring-loaded pins arranged in a hexagonal mesh 3.5 mm apart^59^. In the BLM group, BLM was administered intratumorally (7.5 μg/mouse, 20 μl). In the pIL-12 group, the plasmid IL-12 was injected intradermally into the peritumoral region (4 sites around the tumor, 4 × 20 µl, 0.625 mg/mL, 50 µg). In the combined treatment of the intratumoral BLM injection and application of electrical pulses (ECT BLM), electrical pulses were delivered 2 min after the injection of BLM. In the combined treatment of IL-12 plasmid injection and application of electrical pulses (GET pIL-12), the delivery of electrical pulses was performed immediately after the intradermal injection of IL-12 plasmid in the peritumoral area. In the combined treatment of electrochemotherapy with gene electrotransfer (ECT BLM GET pIL-12) group, the IL-12 plasmid was first administered intradermally in the peritumoral region, and immediately thereafter, electrical pulses were administered as described above for the GET group. Then, 5 min later, BLM was injected intratumorally, and 2 min later, electrical pulses, as for the ECT group, were delivered (Supplementary Fig. S3).

DCE-US examinations were performed in all animals immediately after therapy, 6 h and 1, 3, 7 and 10 days after therapy (Supplementary Fig. S2). SonoVue contrast agent (Bracco, Milan, Italy), a M9 ultrasound machine (Mindray, Shenzhen, China) and a linear probe (L3-13.5, Mindray, Shenzhen, China) with a frequency of 3 to 13.5 MHz and harmonic nonlinear ultraband contrast imaging at a low mechanical index were used. After applying 50 μl of the Alcaine topical ophthalmic anesthetic solution (Alcon, Basel, Switzerland) to the mouse cornea, 0.1 mL of contrast agent was administered into the retro-orbital sinus of the mouse. A 90-s recording was made from the time of contrast agent application. Each tumor was carefully delineated. In addition, 6 to 8 ellipsoidal regions of interest (ROIs) were plotted to cover the entire area of the tumor. For the tumor and ROIs, the perfusion curve or time-intensity curve was analyzed using software integrated in the US device, which displayed the following parameters: BI (base intensity; the baseline intensity when no contrast agent was present), PI (peak intensity), TTP (time to peak; the time at which the contrast intensity reached the peak value), AS (ascending slope; the slope between the starting point of lesion perfusion and the peak value), AT (arrival time; time point at which contrast intensity appeared, generally the time when intensity was 110% higher than the baseline), DT/2 (descend time to half; a time point at which the intensity was half the value of the peak intensity), DS (descending slope) and AUC (area under the curve). PE (peak enhancement) was calculated as the difference between PI and BI (Supplementary Fig. S4). PE and AUC are blood volume parameters, while all others (TTP, AS, AT, DT/2 and DS) describe the blood flow rate. Tumor heterogeneity was assessed using the standard deviation (SD) of parameters for different ROIs of the tumor.

For GET and DCE-US studies, mice were anesthetized under inhalation anesthesia with isoflurane (2% v/v), and heating pads were used to prevent hypothermia.

The weight of the animals was monitored as a sign of systemic toxicity of the treatments. Mice were weighed before the first treatment and then every other day until the end of the experiment.

Tumors were measured every other day using Vernier caliper and tumor volume was calculated from the measured perpendicular diameters (V = a × b × c × π/6). Tumor doubling time (DT) was determined as the time the tumor doubled in volume from the first day of the experiment. Growth delay (GD) was determined as the difference between the tumor doubling time of the individual tumor in the tested group and the mean tumor doubling time in the untreated CTRL group. Mice with tumor regression were examined for the presence of tumors 100 days after treatment. If they were tumor-free 100 days after treatment, then they were considered complete responders (CRs). The results of DCE-US were compared to tumor growth (CR, DT, GD).

In addition, nine mice underwent dynamic contrast-enhanced magnetic resonance imaging (DCE-MRI): two from the CTRL group (on day 1) and seven from the ECT group (immediately, 6, 12, and 36 h after ECT and on day ten in two mice). For DCE-MRI examinations, mice were anesthetized with intraperitoneal application of ketamine (75 mg/kg) and xylazine (3 mg/kg). Gadolinium contrast agent, 100 μL per mouse (gadobutrol 500 mg/kg, Gadovist, 1 mmol/mL (Bayer, Leverkusen, Germany)), was administered via a catheter inserted into the retro-orbital sinus.

### Statistical analysis

One-way analysis of variance followed by the Holm-Sidak test was used to compare differences in tumor growth and DCE-US results among treatment groups (Systat Software, Chicago, IL, USA). Statistical significance was defined as p < 0.05. Daily associations between the PE and (log) DT values or tumor volume and tumor perfusion heterogeneity were examined using linear regression models and are presented in scatterplots with linear regression lines and Pearson correlation analysis. In addition, Bland–Altman plots were used to show differences between DT and PE and the average of these two measurements within each treatment according to the time points of the PE measurements. All analyses and graphical presentations were performed using GraphPad Prism 8.4 (GraphPad software, San Diego, CA).

## Supplementary Information

Supplementary Information.

## Data Availability

The datasets generalised during and/or analysed during the current study are available from the corresponding author on reasonable request.
