# Supplementary material for: Tumor perfusion evaluation using dynamic contrast-enhanced ultrasound after electrochemotherapy and IL-12 plasmid electrotransfer in murine melanoma
Source: Sci Rep. 2021 Jun 29;11:13446. doi: 10.1038/s41598-021-92820-w (PMC8242003; doi:10.1038/s41598-021-92820-w)
Supplement: Supplementary file 1 — Supplementary Information. [file 41598_2021_92820_MOESM1_ESM.docx]

SUPPLEMENTARY INFORMATION

**TUMOR PERFUSION EVALUATION USING DYNAMIC CONTRAST-ENHANCED ULTRASOUND**

**AFTER ELECTROCHEMOTHERAPY AND IL-12 PLASMID ELECTROTRANSFER**

**IN MURINE MELANOMA**

Maja Brloznik^1^, Nina Boc^2^, Maja Cemazar^2,3^,

Gregor Sersa^2,4^, Masa Bosnjak^2^, Simona Kranjc Brezar^2,5*^, Darja Pavlin^1*^

^1^ Clinic for Small Animals, Veterinary Faculty, University of Ljubljana, Gerbičeva 60, Ljubljana, Slovenia

^2^ Institute of Oncology Ljubljana, Zaloška 2, Ljubljana, Slovenia

^3^ Faculty of Health Sciences, University of Primorska, Polje 42, Izola, Slovenia

^4^ Faculty of Health Sciences, University of Ljubljana, Zdravstvena 5, Ljubljana, Slovenia

^5^ Faculty of Medicine, University of Ljubljana; Vrazov trg 2, Ljubljana, Slovenia

**Emails:**

[majabrloznik@gmail.com](mailto:majabrloznik@gmail.com), [nboc@onko-i.si](mailto:nboc@onko-i.si), [gsersa@onko-i.si](mailto:gsersa@onko-i.si), [mcemazar@onko-i.si](mailto:mcemazar@onko-i.si), [mbosnjak@onko-i.si](mailto:mbosnjak@onko-i.si), d[arja.pavlin@vf.uni-lj.si](mailto:arja.pavlin@vf.uni-lj.si), [skranjc@onko-i.si](mailto:skranjc@onko-i.si)

***Corresponding Authors:**

Darja Pavlin, Small Animal Clinic, Veterinary Faculty, University of Ljubljana, Gerbiceva 60, Ljubljana, Slovenia, [darja.pavlin@vf.uni-lj.si](mailto:darja.pavlin@vf.uni-lj.si)

Simona Kranjc Brezar, Institute of Oncology Ljubljana, Zaloska 2, Ljubljana, Slovenia,

[skranjc@onko-i.si](mailto:skranjc@onko-i.si)

**Acknowledgments:** The authors thank Miha Melinec and Tone Jamnik (DIPROS d.o.o.) for their assistance and ultrasonographic machines that enabled contrast studies. The authors acknowledge the financial support of the Slovenian Research Agency (Research Program No P3-003 and No P4-0053). The funder had no influence on the study design, data collection and analysis, the decision to publish, or the preparation of the manuscript.

The language editing of this manuscript was done by American Journal Experts.

**Competing interests:** The authors report no conflicts of interest.

**Authors’ contributions:** Study concepts/study design, S.K.B., D.P., M.C., G.S.; data acquisition, M.B., S.K.B., M.Bo., N.B.; data analysis/interpretation, M.B., N.B., D.P., S.K.B.; manuscript drafting, M.B., D.P., S.K.B.; manuscript revision for important intellectual content, M.C., G.S., S.K.B., D.P.; approval of the final version of submitted manuscript, M.B., N.B., M.C., G.S., M.Bo., D.P., S.K.B.; and manuscript editing, M.B., N.B., M.C., G.S., M.Bo., D.P., S.K.B..

Supplementary Figure and Table Legends

**Supplementary Figure S1:** Dynamic contrast-enhanced magnetic resonance imaging (DCE-MRI) in B16F10 melanoma after electrochemotherapy with bleomycin (ECT BLM). A = untreated control, B = immediately after ECT BLM, C = 6 hours after ECT BLM, D = 36 hours after ECT BLM. To the left, the perfusion curve is presented and to the right, contrast-enhanced T1-weighted image is shown. Note that signal intensity increases for only 70 arbitrary units (a.u.) in B and 110 a.u. in C, while it increases by more than 500 a.u. in A and D.

**Supplementary Figure S2:** Dynamic contrast-enhanced ultrasound (DCE-US) examination of melanoma B16F10; untreated control on day 6: representative images at different times (A = at 3 sec, B = at 6 sec, C = at 20 sec, D = at 40 sec, E = at 80 sec), where the nonlinear contrast mode is shown to the right of each image. Perfusion curves for the whole tumor and different regions of interest (ROIs) are presented in F.

**Supplementary Figure S3:** Schedule for the combined treatment of electrochemotherapy with bleomycin (ECT BLM) and gene electrotransfer of plasmid DNA encoding mouse interleukin-12 (ECT BLM GET pIL-12).

**Supplementary Figure S4:** Schedule for dynamic contrast-enhanced ultrasound (DCE-US) examinations.

**Supplementary Figure S5:** Dynamic contrast-enhanced ultrasound (DCE-US) perfusion curve with a schematic presentation of dynamic parameters. Raw data and fitting curves are presented by M9 (Mindray) ultrasound machine built-in machine software. Peak enhancement (PE) is the difference between peak (PI) and base intensity (BI). The arrival time (AT) is the time after contrast injection until the appearance of contrast. Time to peak (TTP) is the time when the contrast intensity reaches a peak value. Descend time to one-half (DT/2) is the time when the intensity is half the value of the peak intensity. Ascending and descending slopes (AS and DS) refer to slope coefficients. Area under the curve (AUC) is area under the perfusion curve.

**Supplementary Table S1:** Tumor growth delay after electrochemotherapy with bleomycin (ECT BLM) and gene electrotransfer of plasmid DNA encoding mouse interleukin-12 (GET pIL-12) in the melanoma B16F10 model.

**Supplementary Table S2:** Mean peak enhancement (PE) values and their standard error after electrochemotherapy with bleomycin (ECT BLM) and gene electrotransfer of plasmid DNA encoding mouse interleukin-12 (GET pIL-12) in the melanoma B16F10 model. Note that on days 7 and 10, only mice in the therapeutic groups (ECT BLM and ECT BLM combined with GET pIL-12) were measured because mice in the control groups were humanely sacrificed on day 6 due to the disease burden.

**Supplementary** **Table S3:** Pearson correlation coefficients for each day of the dynamic contrast-enhanced ultrasound (DCE-US) measurement, associating peak enhancement (PE) with logarithmically transformed tumor doubling time (DT). Note that data for mice in all groups are presented.

**Supplementary** **Table S4:** Pearson correlation coefficients for each day of the dynamic contrast-enhanced ultrasound (DCE-US) measurement, associating peak enhancement (PE) with logarithmically transformed tumor doubling time (DT). Note that only data for mice in the therapeutic groups (ECT BLM and ECT BLM combined with GET pIL-12) are presented.


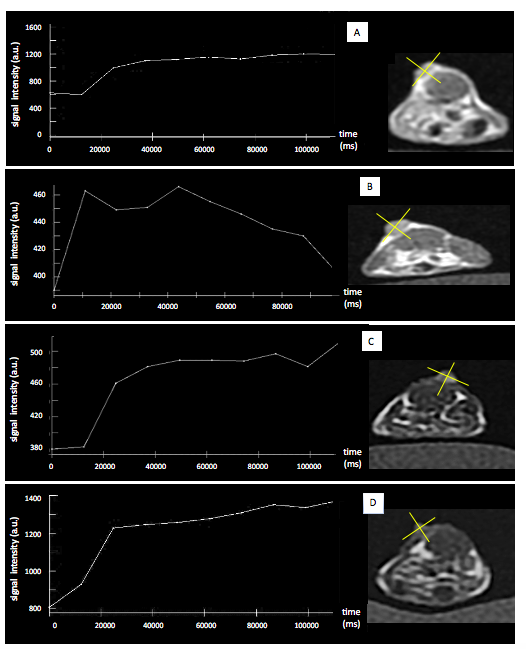


**Supplementary Figure S1:** Dynamic contrast-enhanced magnetic resonance imaging (DCE-MRI) in B16F10 melanoma after electrochemotherapy with bleomycin (ECT BLM). A = untreated control, B = immediately after ECT BLM, C = 6 hours after ECT BLM, D = 36 hours after ECT BLM. To the left, the perfusion curve is presented and to the right, contrast-enhanced T1-weighted image is shown. Note that signal intensity increases for only 70 arbitrary units (a.u.) in B and 110 a.u. in C, while it increases by more than 500 a.u. in A and D.


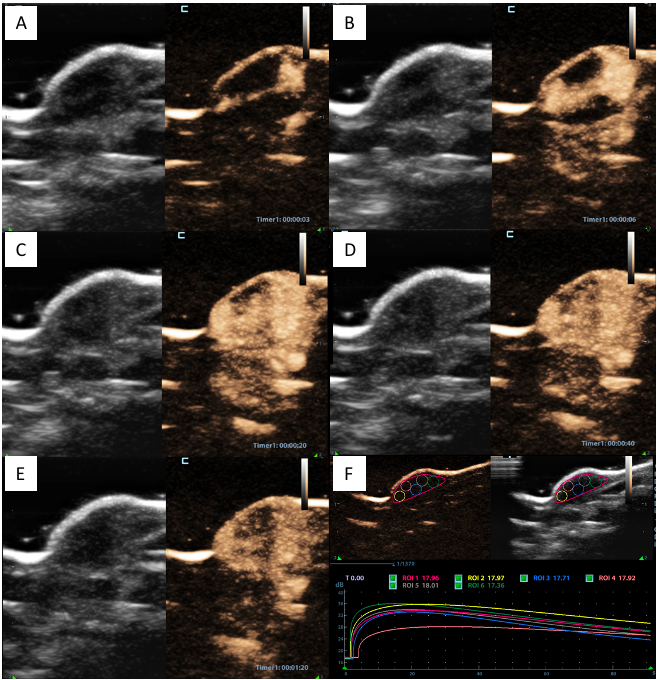


**Supplementary Figure S2:** Dynamic contrast-enhanced ultrasound examination (DCE-US) of melanoma B16F10; untreated control on day 6: representative images at different times (A = at 3 sec, B = at 6 sec, C = at 20 sec, D = at 40 sec, E = at 80 sec), where the nonlinear contrast mode is shown to the right of each image. Perfusion curves for the whole tumor and different regions of interest (ROIs) are presented in F.


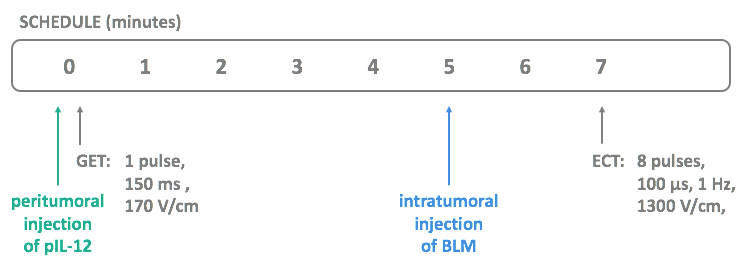


**Supplementary Figure S3:** Schedule for the combined treatment of electrochemotherapy with bleomycin (ECT BLM) and gene electrotransfer of plasmid DNA encoding mouse interleukin-12 (ECT BLM GET pIL-12).


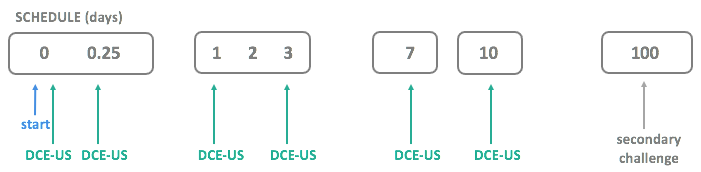


**Supplementary Figure S4:** Schedule for dynamic contrast-enhanced ultrasound (DCE-US) examinations.


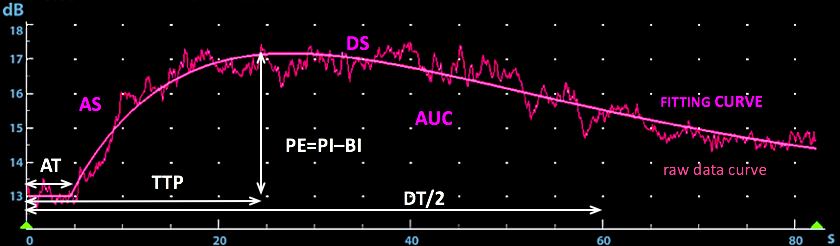


**Supplementary Figure S5:** Dynamic contrast-enhanced ultrasound (DCE-US) perfusion curve with a schematic presentation of dynamic parameters. Raw data and fitting curves are presented by M9 (Mindray) ultrasound machine built-in machine software. Peak enhancement (PE) is the difference between peak (PI) and base intensity (BI). The arrival time (AT) is the time after contrast injection until the appearance of contrast. Time to peak (TTP) is the time when the contrast intensity reaches a peak value. Descend time to one-half (DT/2) is the time when the intensity is half the value of the peak intensity. Ascending and descending slopes (AS and DS) refer to slope coefficients. Area under the curve (AUC) is area under the perfusion curve.

**Supplementary Table S1:** Tumor growth delay after electrochemotherapy with bleomycin (ECT BLM) and gene electrotransfer of plasmid DNA encoding mouse interleukin-12 (GET pIL-12) in the melanoma B16F10 model.

| **Group** | **n** | **DT (days)**  mean±SE | **GD (days)**  mean±SE |
| --- | --- | --- | --- |
| Control | 8 | 1.5±0.2 | / |
| EP (ECT pulses) | 8 | 3.2±0.3 | 1.7±0.3 |
| EP (GET pulses) | 8 | 2.1±0.1 | 0.6±0.1 |
| BLM | 8 | 2.2±0.1 | 0.7±0.1 |
| pIL-12 | 8 | 1.9±0.1 | 0.4±0.1 |
| GET pIL-12 | 8 | 3.4±0.3 | 1.9±0.3 |
| ECT BLM | 8 | 15.5±1.6* | 14.0±1.6* |
| ECT BLM GET pIL-12 | 10 | 28.2±3.6* | 26.7±3.6* |

BLM = bleomycin, 7.5 µg/mouse; ECT = electrochemotherapy; EP = electric pulses; GET = gene electrotransfer; pIL-12 = plasmid DNA encoding mouse interleukin-12; SE = standard error of arithmetic mean; * = p<0.05, statistically significant difference compared to all other group; / = not applicable.

**Supplementary Table S2:** Mean peak enhancement (PE) values and their standard error after electrochemotherapy with bleomycin (ECT BLM) and gene electrotransfer of plasmid DNA encoding mouse interleukin-12 (GET pIL-12) in the melanoma B16F10 model. Note that on days 7 and 10, only mice in the therapeutic groups (ECT BLM and ECT BLM combined with GET pIL-12) were measured because mice in the control groups were humanely sacrificed on day 6 due to the disease burden.

| **Group** | **n** | **PE 0**  **(a.u.)**  mean±SE | **PE 0.25**  **(a.u.)**  mean±SE | **PE 1**  **(a.u.)**  mean±SE | **PE 3**  **(a.u.)**  mean±SE | **PE 7**  **(a.u.)**  mean±SE | **PE 10**  **(a.u.)**  mean±SE |
| --- | --- | --- | --- | --- | --- | --- | --- |
| Control | 8 | 6.5±0.7◎ | 6.5±1.12◎ | 6.4±1.0◎ | 8.9±1.1◎ | / | / |
| EP (ECT pulses) | 4 | 2.1±0.6*♢ | 2.8±0.5✦ | 3.7±0.9 | 6.6±0.9♢✦ | / | / |
| EP (GET pulses) | 4 | 3.8±0.7 | 2.9±0.4 | 2.9±0.5 | 6.3±0.9 | / | / |
| BLM | 3 | 3.8±0.6 | 4.7±1.0 | 3.8±0.6 | 3.6±1.0 | / | / |
| pIL-12 | 4 | 5.8±1.3 | 2.4±1.0 | 4.1±0.6 | 6.0±1.2 | / | / |
| GET pIL-12 | 8 | 3.8±0.8 | 3.7±0.9 | 6.2±1.6 | 4.7±0.9* | / | / |
| ECT BLM | 8 | 2.4±0.8* | 1.6±0.4*⚀ | 2.2±0.3* | 2.2±0.5* | 3.0±0.8 | 5.9±1.3⚀ |
| ECT BLM GET pIL-12 | 8 | 1.7±0.5* | 1.8±0.4* | 2.1±0.6* | 2.5±0.6* | 2.1±0.6 | 2.9±0.5* |

BLM = bleomycin, 7.5 µg/mouse; ECT = electrochemotherapy; EP = electric pulses; GET = gene electrotransfer; pIL-12 = plasmid DNA encoding mouse interleukin-12; PE 0 = peak enhancement immediately after the therapy; PE 0.25 = peak enhancement 6 hours after therapy, PE 1 = peak enhancement 24 hours after therapy, PE 3 = peak enhancement 3 days after therapy, PE 7 = peak enhancement 7 days after therapy , PE 10 = peak enhancement 10 days after therapy, SE = standard error of arithmetic mean; * = p<0.05, statistically significant difference compared to group ◎; / = not applicable, ✦⚀♢ = statistical significance between two groups of mice.

**Supplementary** **Table S3:** Pearson correlation coefficients for each day of the dynamic contrast-enhanced ultrasound (DCE-US) measurement, associating peak enhancement (PE) with logarithmically transformed tumor doubling time (DT). Note that data for mice in all groups are presented.

| day | r | 95% CI | R^2^ | P |
| --- | --- | --- | --- | --- |
| 0 | -0.5871 | -0.7482 to -0.3608 | 0.2492 | ˂0.001 |
| 0.25 | -0.4510 | -0.6535 to -0.1882 | 0.2034 | 0.0015 |
| 1 | -0.4423 | -0.6472 to -0.1777 | 0.1956 | 0.0019 |
| 3 | -0.5976 | -0.7552 to -0.3747 | 0.3571 | ˂0.001 |
| 7 | -0.6067 | -0.8475 to -0.1587 | 0.3681 | 0.0127 |
| 10 | -0.7691 | -0.9157 to -0.4419 | 0.5916 | ˂0.001 |

r = Pearson correlation coefficient, CI = confidence interval, R^2^ = coefficient of determination

**Supplementary** **Table S4:** Pearson correlation coefficients for each day of the dynamic contrast-enhanced ultrasound (DCE-US) measurement associating peak enhancement (PE) with logarithmically transformed tumor doubling time (DT). Note that only data for mice in the therapeutic groups (ECT BLM and ECT BLM combined with GET pIL-12) are presented.

| day | r | 95% CI | R^2^ | P |
| --- | --- | --- | --- | --- |
| 0 | -0.7746 | -0.7482 to -0.3608 | 0.5999 | 0.0004 |
| 0.25 | -0.4729 | -0.7847 to -0.0297 | 0.2237 | 0.0049 |
| 1 | -0.2580 | -0.66683 to -0.2725 | 0.0666 | 0.3346 |
| 3 | -0.5478 | -0.8206 to -0.0715 | 0.3001 | 0.0281 |
| 7 | -0.6067 | -0.8475 to -0.1587 | 0.3681 | 0.0127 |
| 10 | -0.7691 | -0.9157 to -0.4419 | 0.5916 | ˂0.001 |

r = Pearson correlation coefficient, CI = confidence interval, R^2^ = coefficient of determination
